# Interdisciplinarity and Patient Engagement: New Representations of Cardiovascular Anatomy

**DOI:** 10.31083/j.rcm2311366

**Published:** 2022-10-27

**Authors:** Giovanni Biglino, Sofie Layton, Alastair Hamer, Elena Giulia Milano, Massimo Caputo, Jo Wray

**Affiliations:** ^1^Bristol Medical School, University of Bristol, BS8 1TH Bristol, UK; ^2^National Heart and Lung Institute, Imperial College London, SW7 2BX London, UK; ^3^Royal College of Art, SW7 2EU London, UK; ^4^Scuola di Medicina e Chirurgia, University of Verona, 37129 Verona, Italy; ^5^Great Ormond Street Hospital for Children NHS Foundation Trust, WC1N 3JH London, UK

**Keywords:** cardiovascular anatomy, 3D printing, congenital heart disease, aorta, rapid prototyping, patient engagement

## Abstract

**Background:**

This article presents and discusses the genesis, making and public presentation of two artworks by British artist Sofie Layton, namely *Blueprints* and *The Bud*, which explore the anatomy of the heart infusing it with experiential and narrative elements.

**Methods:**

Artist-led workshops with a range of audiences (cardiac patients, medical staff, medical students, creative professionals, and patient relatives) led to explore narratives and imagery that, in turn, was re-presented in artworks exploring the complexity of the cardiovascular system.

**Results:**

While positioning themselves in a long tradition of artistic representations of the heart, often purely anatomical or autobiographical, these artworks stem from a process of patient involvement and participation. Integral to the pieces is an interdisciplinary approach, which is central to arts-and-health collaborations.

**Conclusions:**

At a time in which the role of the arts in improving health and wellbeing is increasingly recognised and supported by evidence, these artworks offer an opportunity to reflect not only on ways of representing cardiovascular anatomy, but also on its experiential value and on the important of patient engagement and involvement.

## 1. Introduction: Representing the Cardiovascular System

The heart is arguably a unique organ in its possessing symbolic, dynamic and musical qualities on top of growth and remodelling properties shared by other organs. Ancient and modern clinicians, thinkers and artists alike have been deeply fascinated by its audible sounds (leading to the heart being represented as musical instruments) or by its symbolic almost mystical dimension (such as in the tradition of religious votive hearts). The connection with the heart, rather than the heart itself as an organ, has also been the subject of representations both anatomical and visceral: from Frida Kahlo’s haunting double portrait *The two Fridas* (1939) and Bill Viola’s video *The science of the heart* (1983) incorporating a surgically dissected heart, to a tradition of artists who have employed their own blood as the ultimate form of self representation, notably Guillermo Gomez-Pena and Ana Mendieyta who later influenced Marc Quinn and Damien Hirst.

Entire books have been dedicated to the subject of the heart’s significance and many meanings across cultures and religions, notably Louisa Young’s *The book of the heart*, to which we will refer again later in the article [1]. A vast body of literature and iconography is dedicated to the cardiovascular system, from ancient Egyptian [2] and Persian [3] societies’ representations and cardiovascular symptoms appearing in Dante’s *Divine Comedy * [4], to seminal books such as Giovanni Battista Morgagni’s* De sedibus et causis morborum per anatomen indagatis* [5] and the wax sculptures created by XVIII century female anatomist Anna Morandi Manzolini at the University of Bologna [6]. Unparalleled anatomical representations of the heart and structures are of course those created by Leonardo da Vinci, including sketches of the coronary vasculature, the cusps of the aortic valve and the sinuses of the aortic root, as well as depictions of phenomena such as vortex formation between the aortic valve cusp and the sinus wall, anticipating hydrodynamic insight into aortic valve closure by nearly four centuries [7]. On the 500th anniversary of his death, Leonardo da Vinci was indeed recognised not only as a visionary inventor and masterful artist, but also as a ‘Renaissance cardiologist’ [7]. More recently, educational initiatives [8] as well as art exhibitions [9] have been dedicated to the human heart. It has been suggested that both historians of cardiology and clinicians should view non-medical representations as a means to appreciate the evolution of their discipline [4] and the preciousness of patients’ experiences [10].

In this light, this article presents a reflection on two artworks stemmed from a participatory process involving cardiovascular patients and created by British artist Sofie Layton as part of *The Heart of the Matter* exhibition (www.insidetheheart.org).

## 2. Methodology 

### 2.1 The Merit of (and Growing Evidence on) the Role of the Arts in Health 

This work can be broadly positioned in the context of arts-and-health collaborations and practices. As a general framework, we refer the reader to the 2019 World Health Organisation scoping review on the global evidence on the role of the arts in improving health and wellbeing [11]. Here, a logic model clearly outlines key components of the arts, namely: aesthetic engagement, involvement of the imagination, sensory activation, evocation of emotion, cognitive stimulation, social interaction, physical activity, engagement with themes of health, interaction with health-care settings. Through psychological, physiological, social and behavioural responses induced by such features of the arts, artistic approaches (in the broadest sense) can impact positively on an array of outcomes, including prevention, management and treatment of disease as well as promoting health behaviours. One of the sub-themes of this analysis particularly discusses that the arts can enhance the understanding of health, thereby improving clinical skills and supporting carers’ wellbeing. Furthermore, the arts can stimulate and facilitate conversations around health. By means of shaping collective thinking, art can contribute to influence and change conversations including on “the forces that shape health” [12], but also captures nuances around narratives of illness and patients’ lived experiences [13].

### 2.2 A Collaborative Approach 

At the very core of the representations of cardiovascular anatomy being discussed in this article, is a profoundly interdisciplinary approach, such as when an artist (SL) and a bioengineer (GB) sat together in front of a computer scrolling through computed tomography (CT) data, with organs appearing and disappearing, and different ways of seeing suddenly coming together. From an engineering perspective, medical imaging data containing 3D information is a precious source for volumetric reconstructions, that in turn can be used for visualising complex structures both virtually and physically, the latter made possible by means of 3D printing technology. From an artistic perspective, the journey inside the body, through the organs, meandering from the ribcage to the spine, in and out, opens a realm of possibilities. In particular, the form (that invisible shape suddenly made visible) can really represent the starting point for the interdisciplinary conversation. While on one level this conversation evolves as ideas are exchanged with a psychologist (JW), a cardiologist (EGM) and a specialist in 3D printing (AH), there is another (profoundly interconnected) level where that same form can be explored, extrapolated and taken beyond its anatomical features in a more emotional and experiential dimension — that of the lived experience, the narrative, the reality of patient’s journey.

### 2.3 A Participatory Approach 

The collaboration underpinning the representation of cardiovascular anatomy was not limited to artist, engineers and medical professionals, but was extended to members of the public (including cardiac patients) through an artist-led participatory process. The participatory element of the work is based on a creative workshop process [14], whereby the artist led groups of participants (group size n = 2–11) through a series of activities including self-portraiture (bi-dimensional by means of blindfolded sketching and three-dimensional by means of modelling clay), creative writing, body mapping and group reflections. The workshop, whilst not overtly medical, included prompts derived from the medical landscape, in the form of MRI-derived heart outlines (that participants could contour and use to develop their own heart-related imagery) and 3D heart models (both realistic as well as artistic renderings including small-scale bronze and silver hearts symbolising the preciousness of the organ). The workshop process was devised either as a one-day or two-day experience and offered to a range of participants, including cardiac patients (e.g., valvular heart disease, congenital heart disease, arrhythmias, heart transplantation), medical staff (e.g., cardiologists, imagers, nurses, anatomists, general practitioners), medical students, creative professionals (e.g., writers, visual artists, musicians, poets, museum staff) and patient relatives. All participants provided informed consent prior to taking part in the workshop process. By the end of the workshop, participants were invited to develop imagery and metaphors related to their heart – how they perceive it, what they think it looks like, what it represents to them. After approximately 100 workshop interactions, a collection of narratives and images was collected, including some unique imagery as well as imagery that recurred across different groups. Thereafter, as part of a process of artistic re-presentation and filtering of such imagery, the lead artist selected fourteen such images that resonated particularly across all groups and, working across different media, gave form to them in a series of original artworks and installations that in the end resulted in *The Heart of the Matter* exhibition. Here we discuss two of such artworks.

## 3. Results

### 3.1 Two-Dimensionality: The Plan of the Engine Room 

**Fig. 1.**
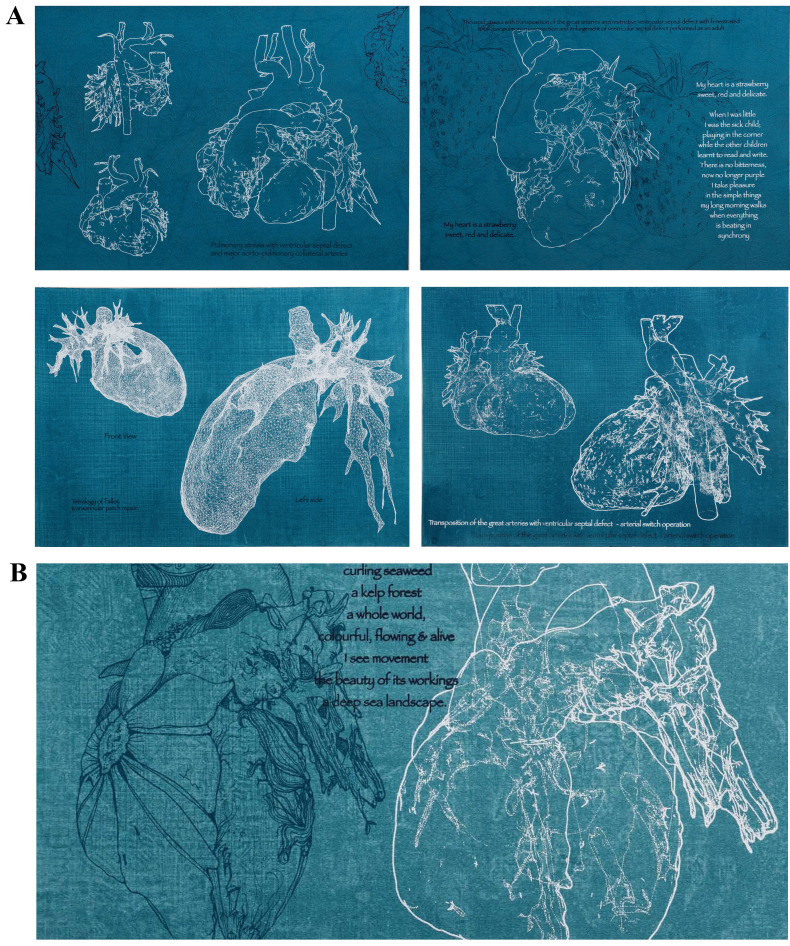
***Blueprints* - screen prints on canvas (series), by Sofie Layton**. The blueprints explore the architectural quality of the heart derived from modelling of patients’ hearts and representing a wide range of ages and conditions (A). Sometimes a blueprint is accompanied by a more poetic annotation from the person whose heart is represented (B), other times it is pure line. Image from [15] with permission.

**Fig. 2.**
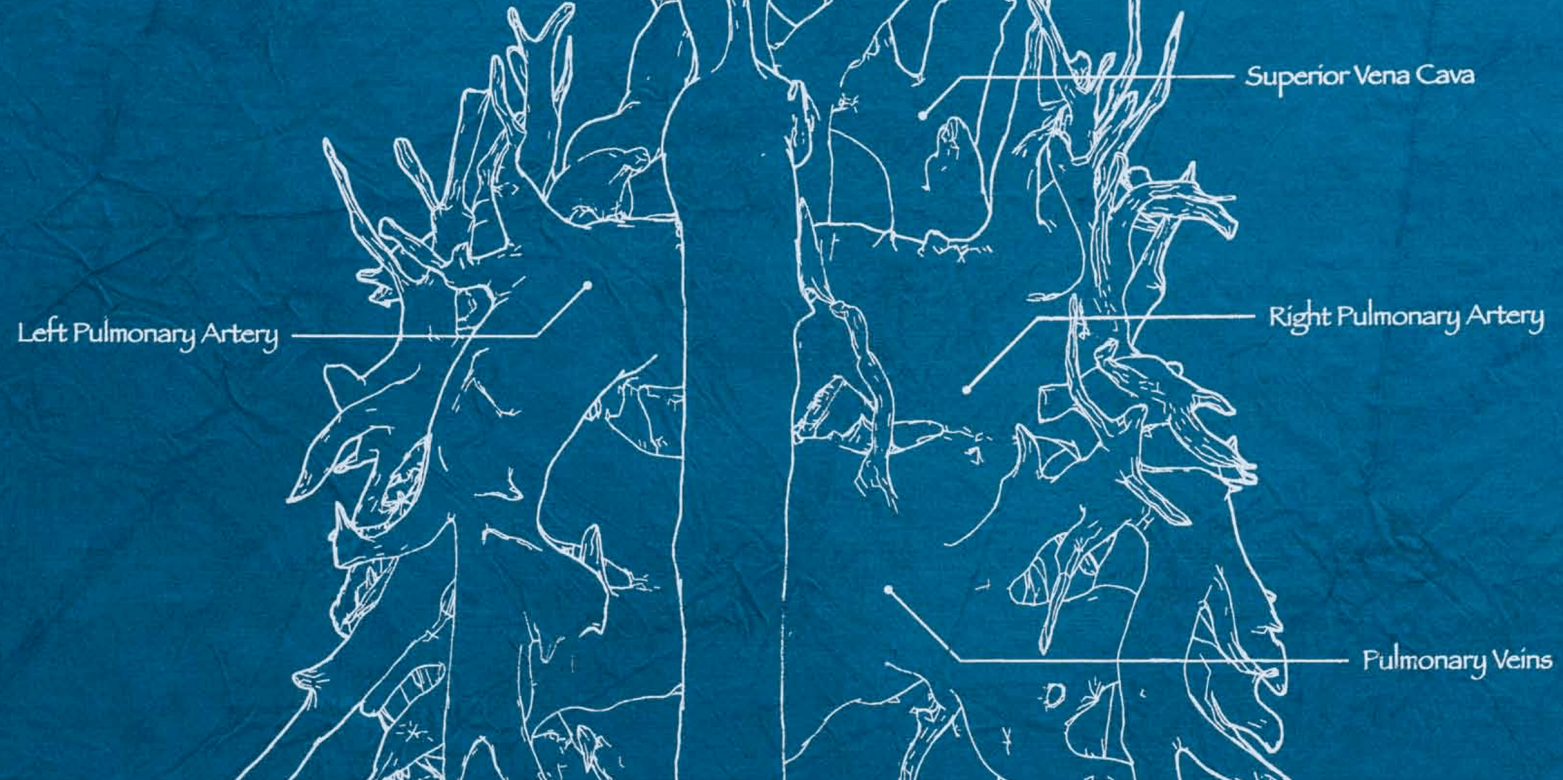
**Example of anatomical annotations on one of the *Blueprints *(detail)**. Image from [15] with permission.

The heart seen as the engine room of the human body, the fundamental motor, the source of energy, the incessant rhythmical pump, lent itself to explore the visual language of architectural drawings. What is the structure of this room or device? Can its complexity be sketched in its most fundamental lines and contours? Exploring such visual narrative, the artist created a set of 27 screen prints on canvas, titled *Blueprints*, displayed in different multiples of 9 depending on the setting (Fig. 1, Ref. [15]). The textile pieces (50 × 70 cm) evoke the architectural sketches known as ‘blueprints’ referencing the characteristic white lines on a blue background typical of the cyanotype process. From an artistic standpoint, the choice of a blue background with white outlines is a reference to the form of photography known as ‘cyanotypes’, introduced in 1842 by Sir John Herschel and characterised by the Prussian blue tonality achieved by treating the paper with an iron-salt solution [16], a process that went on to be used extensively in architectural drawing. Evoking thus the idea of an architectural sketch, the series overtly focuses on the structure, the outlines, the form of the heart. Here, the white lines are those of patient-specific MRI-derived outlines. These were derived first creating a 3D file from the MRI data with commercial software (Mimics, Materialise LV, Leuven, Belgium) and then further processing such stereolithography (.stl) files extrapolating their surface lines in a different package (Rhino, Robert McNeel & Associates, Seattle, WA, USA). The anatomy of hearts with different defects (e.g., transposition of the great vessels, tetralogy of Fallot, tricuspid atresia, pulmonary atresia) are presented from different views and labelled (e.g., top view, front view, left side etc.). The labelling itself is part of the language that the artist willingly explores in the piece, including on the one hand the technical anatomical terminology (in some cases labelling different blood vessels, Fig. 2 (Ref. [15])) and on the other hand incorporating the patients’ own descriptions using their own words. “My heart is a strawberry, sweet, red, and delicate”. “My heart is a kelp forest”. These very personal simple metaphors are interwoven with the accurate anatomical representation and in some cases they are accompanied by a delicate drawing, in a darker almost faint line, representing the more personal description alongside the anatomical sketches. Can the two really be told apart?

### 3.2 Three-Dimensionality: A Growing Arterial Tree 

**Fig. 3.**
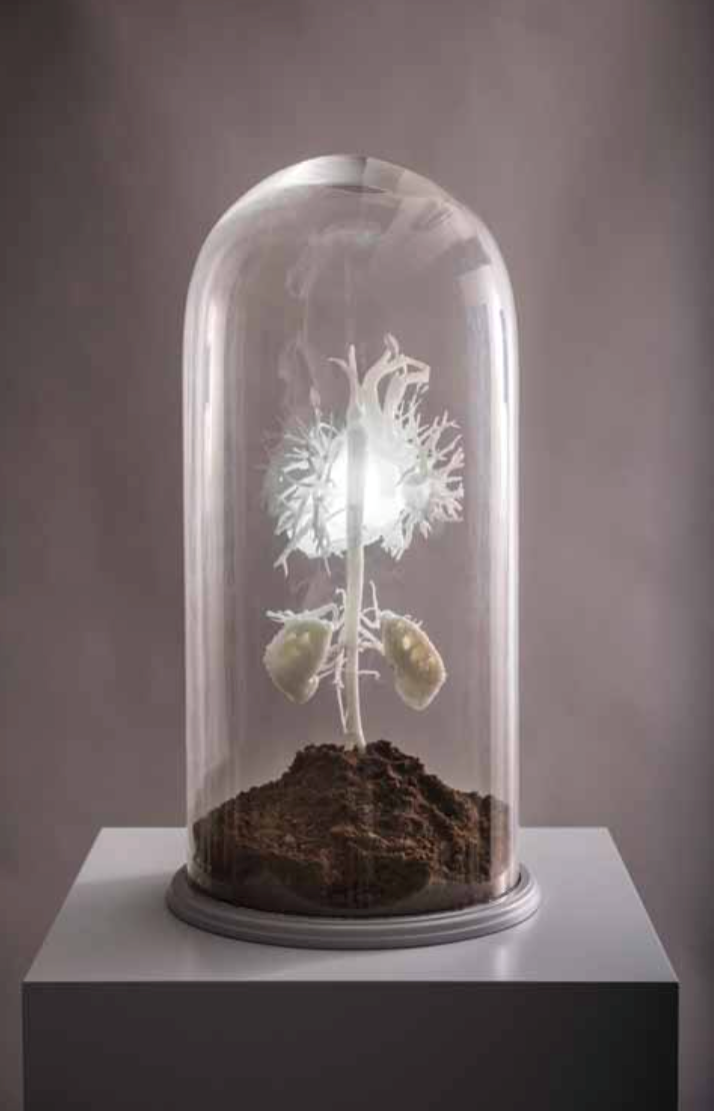
***The bud* - 3D printed model of heart, vasculature and kidneys in 1:1 replica, by Sofie Layton**. Image from [15] with permission.

The 3D complexity of the heart is instead explored in a sculptural piece, titled *The bud* (Fig. 3, Ref. [15]). Here the artist draws on a recurrent theme emerged during the workshop process, whereby participants describe their hearts as living organisms, plants, trees, or flowers. Rooting again the piece in scientific/medical language, the sculpture is a 1:1 model of a heart including the aorta to the level of the iliac bifurcation, the main vessels (brachiocephalic, pulmonary, splanchnic) and the kidneys. The MRI-derived 3D model was 3D printed as a hollow structure in white resin using the Objet Polyjet process. The artist then configured the model as emerging from soil under a huge glass bell jar, and illuminated the model by advancing LED lights inside the model itself. The overt botanical reference is subtle — just a few white leaves made out of fabric are stemming from some of the blood vessels — but the configuration is such that the arterial tree looks both protected and alive under its bell jar, with the temperature from the lights resulting in condensation on the glass conferring another layer of living (almost breathing) quality to the piece. *The bud* positions itself in a long history of representations that draw on the branching, arboreal quality of the vascular system. As reported by Louisa Young, the allegory of the tree is an ancient one [1]: knowledge of branches stemming from the heart anticipated the full appreciation of the cardiovascular network, and Young cites Aristotle to this end (“From the heart springs out an artery as does the trunk of a tree from the earth [and] just like the trunk of a tree divides into branches”). The image is reprised across the centuries (notably Leonardo da Vinci suggested the similarity between the heart and the seed, in a 1504-6 drawing in the Royal Collection: “The plant first exists before the branches and the heart exists before the veins”) and across cultures (the Tree of Life from the Cabbala, the chakras in yoga, the XII century Book of Bahir) [1]. Historically, the botanical allegory includes the heart as a rose (charged with religious symbolism) or as a lotus (in Hinduism and Buddhism), or different fruits (from the *Madonna with pomegranate* by Botticelli to the Christ holding grapes in Juan Correa’s *Allegory of the Sacrament*) [1]. Whilst confirming the relevance and timelessness of an ancient allegory, *The bud* interestingly stemmed from a process that focused on exploring individual uniqueness [14], and only in the end invited participants to explore more explicitly the representation of their heart, ultimately representing to some extent a collective narrative, rather than an individual (often autobiographical) representation, as it is the case in several contemporary artistic representations.

## 4. Discussion: The Value of Interdisciplinarity 

These artworks allow for a broader reflection in terms of how today we can explore, represent and discuss cardiovascular anatomy (and arguably our anatomy in general).

First and foremost, incorporating patient experience and language and individuals’ views into the artworks themselves results in stimulating and thought-provoking layering; the artwork can be entered in different ways, it can be seen from different angles, focusing on one element (e.g., medical vs non-specialist language, anatomical detail, metaphorical elements) but also reflecting on the experience(s) behind the piece. Whether individual, collective or composite [17], the narrative element adds the experiential and societal dimensions to illustrative, didactic and even autobiographical possibilities. Literature has also importantly shown that patients’ own representations are linked to functional outcomes. A study focusing on patients’ drawings of damage to their hearts following myocardial infarction showed that patients’ representations predicted recovery better than medical indicators of damage did [18], highlighting that drawings provided the clinician with valuable entry points to explore patients’ ideas and perceptions of their own cardiovascular health, including potential negative illness beliefs that could subsequently be discussed and rectified.

Secondly, technology allows for new possibilities bridging the worlds of biotech and art-making, whereby 3D models used within a medical study [19] can be presented in a creative context or even incorporated in an installation [20, 21]. Such a technology enables the artist to explore ideas around making the invisible visible and the invisible tangible which is artistically fascinating as it gives a form to this unique organ. We feel its presence in our body and metaphorically use it to describe our emotional experience of the world, but medical imagining data bring us conceptually closer to our connection to the heart and its inner workings.

Thirdly, the interdisciplinary collaboration underpinning the genesis, the making and the presentation of these pieces is exciting because it magnifies the opportunities of exploration and expression by virtue of bringing together different languages, different points of view, different ways of seeing when dealing with something as complex as cardiovascular anatomy or indeed when considering narratives of illness more broadly. It is a delicate balance, as the artist’s vision ultimately should not be curtailed but rather enriched by reflections and exchanges across the interdisciplinary team. This quality of the work is like an undercurrent throughout its stages, whether discussing technical elements of the printing of the 3D models or annotating the blueprints with appropriate medical language or planning with the psychologist the most appropriate way to share the work with the workshop participants in a way that is both sensitive and enriching.

What happens when the work is presented to an audience should finally be considered, in terms of its ramifications, its potential impact as a catalyst for reflections and conversations and a means of expression to honour someone else’s story. As mentioned, the role of the arts in improving health and wellbeing is increasingly recognised and supported by evidence, and the recent WHO scoping review [11] outlines aesthetic engagement, involvement of the imagination and evocation of emotion amongst key components leading to psychological, physiological, social and behavioural responses ultimately impacting on health-promoting behaviours and on prevention, management and treatment of disease. Considerations on the public presentation of artworks stemming from a participatory approach and imbued with patients’ experiences are therefore very important (and beyond the scope of this reflection). But we would like to conclude by considering the significance of sharing such work within a medical audience specifically. The abovementioned importance of viewing non-medical representations for clinicians to gain a different perspective on their discipline could perhaps even be extended to a potential role within evidence-based medicine. It has been suggested that evidence-based medicine can be unintentionally affected by biases impacting negatively on the healthcare agenda, including assigning a low status to experience in the evidence hierarchy and suppressing the patient’s voice [22]. Whilst posing questions as to how to demonstrate rigorously such potentially very meaningful impacts, these considerations only reiterate the importance of patient involvement in research, including in arts-and-health collaborations, which can in fact represent a model of interdisciplinarity.

Whilst it is recognised that different audiences may respond differently to different elements of a piece (e.g., academic audience vs. clinical audience vs. general audience), the learning from the evaluation itself is always important. In the authors’ experience, for instance, an immersive installation including anatomical representations of heart disease and stories narrated by patients’ voices led to an empathic response and an appreciation of the value of illness narratives [20]. A more general recent analysis on the role of representations of cardiovascular health and disease (including novels, films and paintings) concluded that such artistic representations (i) reflect cognitions of disease, (ii) can shape views of ill and healthy individuals with respect to heart diseases, and (iii) can thus contribute to improving quality of life of cardiovascular patients [23]. In agreement with this study and in recognition of their beneficial role, we conclude that artistic representations of disease warrant further collaborative research.

## 5. Conclusions

Evaluating audiences’ responses to artworks stemming from participatory processes is extremely important not only to contribute to the abundant and growing evidence on the role of the arts in health, but also to inform future artistic interventions and collaborative projects.
